# A convolutional neural network-based model that predicts acute graft-versus-host disease after allogeneic hematopoietic stem cell transplantation

**DOI:** 10.1038/s43856-023-00299-5

**Published:** 2023-05-16

**Authors:** Tomoyasu Jo, Yasuyuki Arai, Junya Kanda, Tadakazu Kondo, Kazuhiro Ikegame, Naoyuki Uchida, Noriko Doki, Takahiro Fukuda, Yukiyasu Ozawa, Masatsugu Tanaka, Takahide Ara, Takuro Kuriyama, Yuta Katayama, Toshiro Kawakita, Yoshinobu Kanda, Makoto Onizuka, Tatsuo Ichinohe, Yoshiko Atsuta, Seitaro Terakura

**Affiliations:** 1 Department of Hematology and Oncology, Graduate School of Medicine, Kyoto University, Kyoto, Japan; 2 Center for Research and Application of Cellular Therapy, Kyoto University Hospital, Kyoto, Japan; 3 Department of Hematology, Hyogo Medical University Hospital, Hyogo, Japan; 4 Department of Hematology, Federation of National Public Service Personnel Mutual Aid Associations Toranomon Hospital, Tokyo, Japan; 5 Hematology Division, Tokyo Metropolitan Cancer and Infectious Diseases Center, Komagome Hospital, Tokyo, Japan; 6 Department of Hematopoietic Stem Cell Transplantation, National Cancer Center Hospital, Tokyo, Japan; 7 Department of Hematology, Japanese Red Cross Aichi Medical Center Nagoya Daiichi Hospital, Nagoya, Japan; 8 Department of Hematology, Kanagawa Cancer Center, Yokohama, Japan; 9 Department of Hematology, Hokkaido University Hospital, Sapporo, Japan; 10 Department of Hematology, Hamanomachi Hospital, Fukuoka, Japan; 11 Department of Hematology, Hiroshima Red Cross Hospital & Atomic-bomb Survivors Hospital, Hiroshima, Japan; 12 Department of Hematology, National Hospital Organization Kumamoto Medical Center, Kumamoto, Japan; 13 Division of Hematology, Jichi Medical University Saitama Medical Center, Saitama, Japan; 14 Department of Hematology/Oncology, Tokai University School of Medicine, Isehara, Japan; 15 Department of Hematology and Oncology, Research Institute for Radiation Biology and Medicine, Hiroshima University, Hiroshima, Japan; 16 Japanese Data Center for Hematopoietic Cell Transplantation, Nagoya, Japan; 17 Department of Registry Science for Transplant and Cellular Therapy, Aichi Medical University School of Medicine, Nagakute, Japan; 18 Department of Hematology and Oncology, Nagoya University Graduate School of Medicine, Nagoya, Japan

**Keywords:** Haematopoietic stem cells, Computational biology and bioinformatics, Haematological diseases, Graft-versus-host disease

## Abstract

**Background:**

Forecasting acute graft-versus-host disease (aGVHD) after allogeneic hematopoietic stem cell transplantation (HSCT) is highly challenging with conventional statistical techniques due to complex parameters and their interactions. The primary object of this study was to establish a convolutional neural network (CNN)-based prediction model for aGVHD.

**Method:**

We analyzed adult patients who underwent allogeneic HSCT between 2008 and 2018, using the Japanese nationwide registry database. The CNN algorithm, equipped with a natural language processing technique and an interpretable explanation algorithm, was applied to develop and validate prediction models.

**Results:**

Here, we evaluate 18,763 patients between 16 and 80 years of age (median, 50 years). In total, grade II–IV and grade III–IV aGVHD is observed among 42.0% and 15.6%. The CNN-based model eventually allows us to calculate a prediction score of aGVHD for an individual case, which is validated to distinguish the high-risk group of aGVHD in the test cohort: cumulative incidence of grade III–IV aGVHD at Day 100 after HSCT is 28.8% for patients assigned to a high-risk group by the CNN model, compared to 8.4% among low-risk patients (hazard ratio, 4.02; 95% confidence interval, 2.70–5.97; *p* < 0.01), suggesting high generalizability. Furthermore, our CNN-based model succeeds in visualizing the learning process. Moreover, contributions of pre-transplant parameters other than HLA information to the risk of aGVHD are determined.

**Conclusions:**

Our results suggest that CNN-based prediction provides a faithful prediction model for aGVHD, and can serve as a valuable tool for decision-making in clinical practice.

## Plain language summary

Hematopoietic stem cell transplantation (HSCT) is a procedure used in patients to reestablish blood cell production. It involves the transplant of cells from a donor to the patient. In some patients the transplanted cells damage cells within the patients. This is called graft-versus-host disease (GVHD). We developed a computational code that can predict the likelihood a person will develop GVHD soon after HSCT. Using this computer program will enable doctors to better identify those at risk of GVHD and initiate treatments when required.

## Abstract (web summary)

Jo et al. establish a convolutional neural network-based model to predict acute graft-versus-host disease (aGVHD) after allogeneic hematopoietic stem cell transplantation. The model both predicts aGVHD and identifies pre-transplant parameters that increase risk of aGVHD.

## Introduction

Allogenic hematopoietic stem cell transplantation (HSCT) can be a curative therapeutic procedure for malignant or nonmalignant hematological diseases. Even though transplantation outcomes have improved in recent years^1^, the incidence of transplant-related mortality (TRM) remains as high as 30% among HSCT recipients^2^. Acute graft-versus-host disease (aGVHD) is one of the most critical complications after HSCT and can lead to TRM^3^. Therefore, accurate prediction of the risk of developing aGVHD is essential to reduce the risk of TRM by optimizing donor selection and transplantation procedures.

Most previous studies on risk assessment of aGVHD have employed conventional linear proportional hazard models, including the Cox proportional hazard^4,5^. However, such models are simplified by assuming that the log-risk function is linear. Importantly, these studies suffered limitations due to the arbitrary setting of variables. For example, the human leukocyte antigen (HLA) disparity between donors and recipients has been treated as a binary variable, i.e., matched or mismatched; however, the degree of HLA disparity may not be equivalent, depending on the combinations with alleles between different HLA loci and the combination of specific HLA antigens or alleles between donor and recipient^6^. Thus, HLA information should be handled as close to raw data without arbitrariness. Moreover, while previous studies have focused on HLA mismatches, the extent to which various factors other than HLA affect the risk of developing aGVHD, or whether they do at all, has remained murky due to highly complex clinical parameters and their interactions. Therefore, conventional models do not predict the occurrence of aGVHD reliably enough to be applied to individual cases in clinical practice.

Recent application of machine learning algorithms, which perform statistical calculations without the assumptions required by conventional methods, is beginning to provide novel insights into clinical practice^7–9^. However, these previous studies utilizing machine learning algorithms have not solved the arbitrariness of variable settings and failed to incorporate detailed, raw clinical data. Moreover, many machine learning-based models do not explain the model’s learning process, nor do they indicate why the model predicted that specific cases would develop aGVHD. This “black-box” nature of machine learning constitutes a barrier to the implementation of machine learning-based models in clinical practice^10^.

Among various machine-learning methods, convolutional neural networks (CNNs) are promising machine-learning algorithms that excel at feature extraction; thus, they are well suited to overcome the limitations of existing methods^11^. The CNN-based method has an affinity for natural language processing, which can automatically convert complex information in a database into a computer-friendly representation. This feature is advantageous for modeling HSCT because HLA information is close to natural language from the viewpoint of data processing. Moreover, CNN is able to visualize learning processes that help clinical decision-making^12^. Nonetheless, the usefulness of CNN-based prognostic prediction in HSCT has not been evaluated yet.

Thus, in this study, we applied CNN algorithms to develop a prediction model for aGVHD after HSCT, which can incorporate detailed raw HLA information, as well as various non-HLA variables, and can transparently visualize the contribution of each variable in the learning process. This prediction model revealed that the risk of aGVHD is determined not only by HLA disparity but also by detailed HLA information, as well as various clinical factors other than HLA. We expect our results to provide a clinically useful model for predicting aGVHD risk, and to offer insights into the complex decision-making process of a machine-learning system in the field of transplantation.

## Methods

### Patient inclusion and exclusion criteria

Data on adult patients (age ≥16 years) with malignant and nonmalignant hematological diseases who underwent allogeneic HSCT between 1 January 2008 and 31 December 2018 were obtained from the Japanese Transplant Registry Unified Management Program^13,14^, sponsored by the Japanese Society for Transplantation and Cellular Therapy (JSTCT) and the Japanese Data Center for Hematopoietic Cell Transplantation. Patients were excluded if information about HLA mismatch, aGVHD grade, or clinical outcomes (dead or alive) was missing. Our protocol, which complied with the Declaration of Helsinki, was approved by the Ethics Committee of Kyoto University and the Japanese Data Center for Hematopoietic Cell Transplantation. Patient information is anonymized, and patients consented to provide their data to the data center prior to the initiation of the study.

### Data collection and definition of each covariate

From the registry database, we extracted data on all pre-transplant characteristics (Supplemental Data 1), along with data on post-transplant aGVHD grade and prognoses. Patients were divided into standard- and advanced-risk groups according to previous criteria for determining disease risk^15,16^. Eastern cooperative oncology group performance status scale (ECOG PS) at transplantation was evaluated according to ECOG criteria^17^. Major organ complications were assessed using hematopoietic cell transplantation-specific comorbidity index (HCT-CI) according to the Seattle scale^18^. Conditioning intensity was defined according to operational definitions of the National Marrow Donor Program/CIBMTR^19^. GVHD prophylaxis was performed at the discretion of the institutions, and in the majority of cases, a combination of either cyclosporin A (CyA) or tacrolimus (Tac) with methotrexate (MTX) or mycophenolate mofetil (MMF) was adopted. Disparities in HLA-A, HLA-B, and HLA-DR antigens were determined at the serologic level from relatives and cord blood transplants. In unrelated bone marrow and peripheral blood stem cell transplants, 8 antigens, including HLA-C, were examined at the allele level. A 6/6 or 8/8 match was considered HLA matched^2,20^. Diagnosis and classification of aGVHD cases were performed by the attending physicians at each center based on conventional criteria^21^.

### Development of a prediction model of aGVHD based on CNN algorithms

Predictive models for aGVHD (grade II–IV and III–IV) were developed using CNN algorithms. The CNN architecture was implemented in Python using the Keras library^22^, which is a high-level library for TensorFlow version 2.2ML framework^23^. The CNN architecture included an input layer, a modified bottleneck layer, a global average pooling layer, fully connected (FC) layers, and output layers. The CNN model took inputs from patient data, where HLA information was pre-processed using word2vec^24^, a natural-language processing (NLP) application, in which antigens and alleles of HLA-A, B, -C, and DRB1 in both recipient and donor were treated as words that generate vectors. In the modified bottleneck layer, there were short-cut connections that skip indicated layers, and ResNet, a residual learning framework was used to optimize and train the deep networks^25^. *L2* regularization was adopted to avoid over-fitting, thereby ensuring the availability of the proposed architecture. *Adam* was chosen as the optimizer to compute different and adaptive learning rates for each parameter using a batch size of 32 for an initial learning rate of 0.01 with a decay rate of 0.9. We randomly split the whole cohort into 65%, 15%, and 20% sub-cohorts for training, validation, and testing purposes, respectively. The training set was used to train the network, and learnable parameters were updated via backpropagation. The validation set was employed to monitor the model’s performance during the training process, thereby establishing the reliability of learning results. In order to evaluate the generalizability of the CNN algorithm, the test set was used to assess the efficacy of a trained model on data that it had not seen previously.

### t-Distributed stochastic neighbor embedding (t-SNE)

t-SNE is a dimensionality reduction technique that allows high-dimensional data to be mapped in two dimensions and visualized as a scatter plot^26^. In this study, t-SNE was adapted to reduce the dimensions of the distributed representation in the neural network algorithm, including word embedding space, thereby visualizing word embedding of HLA information and features of the indicated layers of the model. We employed t-SNE plots of individual patients using pairwise distances in high dimensions. This means that each plot is equal to one patient. In general, patients closest to each other are most similar, while those farthest apart are most different. On all t-SNE maps the axes are called t-SNE dimension 1 and t-SNE dimension 2 to show the separation of risk scores in the test dataset. These axes lack concrete meaning themselves due to the technical nature of t-SNE method. Higher scores are associated with higher incidences of aGVHD.

### Local interpretable model-agnostic explanations (LIME)

We used LIME^27^ to explain predictions from the CNN algorithm. LIME is a local linear approximation of the model’s behavior. While CNN is complex globally, it is easier to approximate it close to the neighborhood of a particular observation. By stimulating other observations around that observation, LIME fits a sparse linear model in this local region to assess the positive and negative effects of each predictor in the CNN to estimate the incidence of aGVHD. It provides both an explanation of an instance by an interpretable representation as well as visualization. The lime R package (https://cran.r-project.org/web/packages/lime/) was used to perform the analysis.

### Clinical evaluation of the generalizability of the trained model

Assessment of the generalizability of the developed CNN-based model was performed using the test cohort (comprising 20% of the entire cohort) using conventional statistical methods. To assess whether the developed model could identify patient populations at extremely high or low risk of aGVHD, we divided the cohort into three groups according to percentile scores for grade II–IV and grade III–IV aGVHD: low-risk group (Low; 0–10th percentile), intermediate-risk group (Int;10th–90th percentile), and high-risk group (High; 90th–100th percentile). Overall survival (OS) was calculated using the Kaplan–Meier method and compared using the Cox proportional-hazards model according to aGVHD predictive scores determined by the CNN algorithm. The cumulative incidence of aGVHD was calculated using Gray’s method while considering relapse and death as competing risks^28^. The Fine-Gray proportional-hazards model was used to compare the incidence of aGVHD with aGVHD predictive scores that were determined by the CNN-based model^29^. TRM was calculated considering relapse as a competing risk^30^. Stata (version 17; Stata Corp., College Station, TX) was used to analyze data. *p* < 0.05 was considered statistically significant.

### Reporting summary

Further information on research design is available in the Nature Portfolio Reporting Summary linked to this article.

## Results

### Patient characteristics

We evaluated 18,763 patients between 16 and 80 years of age (median, 50 years), who underwent allogeneic HSCT between 2008 and 2018 (Supplemental Data 2). The most common indication of HSCT was acute myeloid leukemia (AML) or myelodysplastic syndrome (MDS) (*n* = 10,780, 57.5%) followed by acute lymphoblastic leukemia (ALL) (*n* = 3609, 19.2%). Graft sources were related bone marrow (BM) in 1803 cases (9.6%), related peripheral blood stem cells (PBSC) in 3993 (21.3%), unrelated BM in 7232 (38.5%), unrelated PBSC in 403 (2.2%), and unrelated cord blood (CB) in 5332 (28.4%). HLA-matched donors were selected in 10,131 cases (54.0%), and HLA-mismatched donors were selected in the remaining 8632 (46.0%). The median follow-up period for survivors was 45.4 months after HSCT. In total, grade II–IV aGVHD was observed among 42.1% of all patients (*n* = 7895) on Day 30 in the median after HSCT, while grade III–IV aGVHD was noted in 15.6% of all patients (*n* = 2930) on Day 33 in the median after HSCT. Severe aGVHD (grade III–IV) resulted in TRM in 45.4% of patients with grade III–IV aGVHD (*n* = 1329). Overall, 377 of these patients died of aGVHD (12.9%).

### Development of CNN-based prediction models for aGVHD

**Fig. 1:**
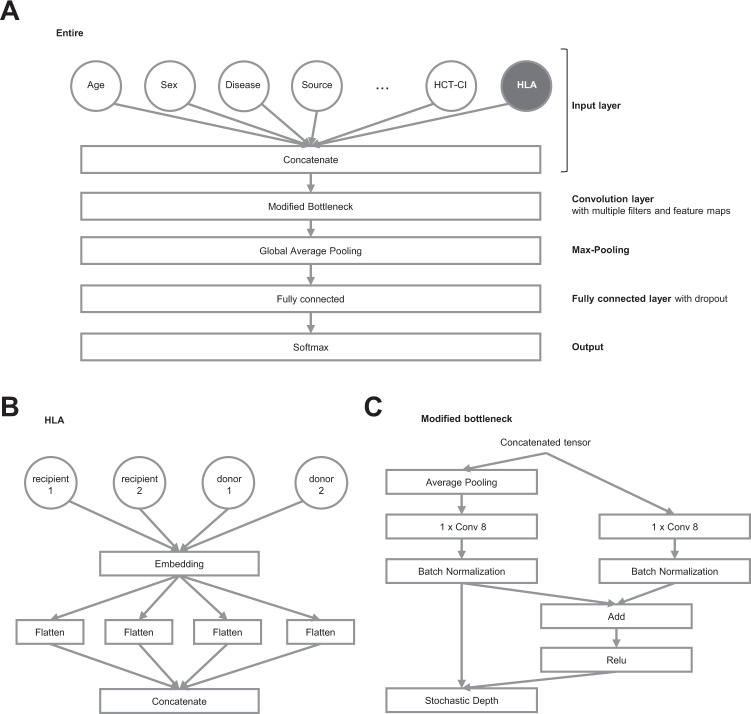
Convolutional neural network (CNN) structure for prediction of grade II–IV and grade III–IV acute graft-versus-host disease (aGVHD). **A** The entire structure of the CNN model. HLA information (shown in gray-filled, blanked letters) processed with natural language processing. **B** Processing of HLA information. **C** The detailed structure of the modified bottleneck part.

We randomly split the cohort into 65%, 15%, and 20% for training, validation, and testing purposes, respectively. There were no significant differences among these sub-cohorts in terms of pre-transplant characteristics (Supplemental Data 2). Predictive models for grade II–IV and III–IV aGVHD were developed utilizing CNN-based models with the training and validation cohorts. As a result, the final CNN architecture included an input layer, a modified bottleneck layer, a global average pooling layer, fully connected (FC) layers, and an output layer (Fig. 1A). The CNN model took inputs from patient data (Supplemental Data 1), and HLA information was pre-processed using word2vec (Fig. 1B). In the modified bottleneck layer, there were short cut connections that skip indicated layers, and ResNet, a residual learning framework^25^, was used to ease the training process of deep networks (Fig. 1C). The learning process with the CNN-based model eventually allowed us to calculate a prediction score for grade II–IV or III–IV aGVHD for individual cases, which predicts the risk of developing grade II–IV or III–IV aGVHD (Supplemental Fig. 1A and B).

### Visualization of the learning process

**Fig. 2:**
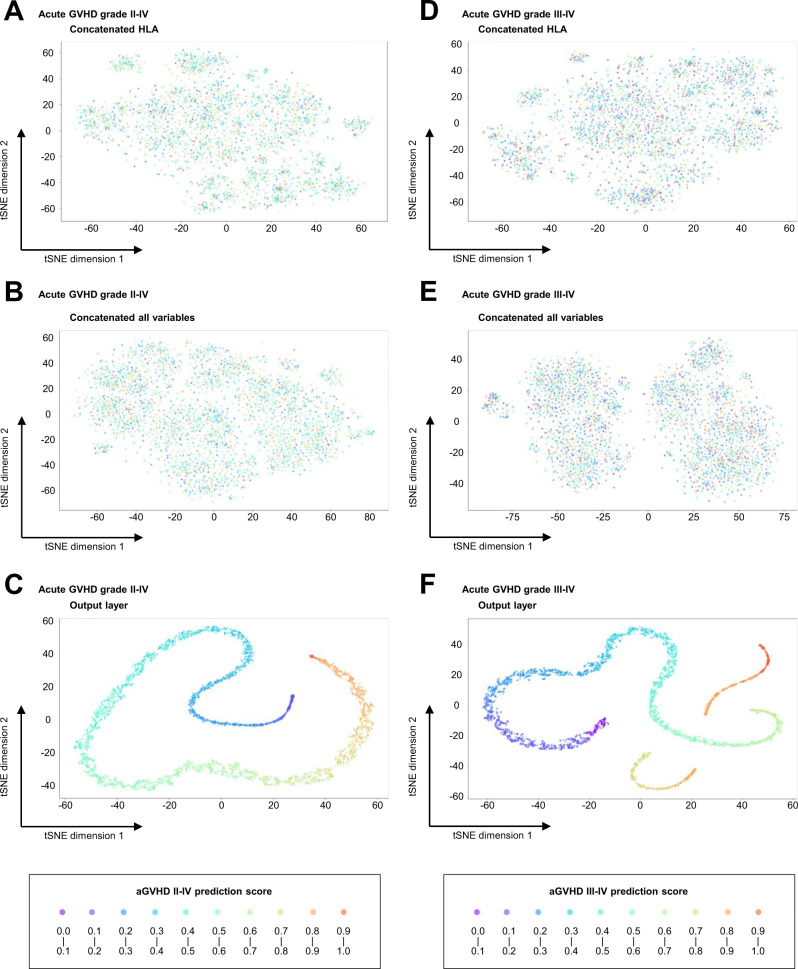
t-Distributed stochastic neighbor embedding (t-SNE) visualization of the output of each layer in the convolutional neural network (CNN). **A–C** t-SNE for grade II**–**IV acute graft-versus-host disease (aGVHD). **D–F** t-SNE for grade III**–**IV acute graft-versus-host disease (aGVHD). **A**, **D** Outputs after concatenating HLA information in the input layer. **B**, **E** Outputs after concatenating all variables in the input layer. **C**, **F** Outputs in the output layer. Each plot is equal to one patient. In general, patients closest to each other are most similar, while those farthest apart are most different.

In order to show how input data is transformed through each layer, we visualized outputs of hidden layers that were reduced to two dimensions using t-Distributed Stochastic Neighbor Embedding (t-SNE) in the CNN model for grades II–IV and III–IV aGVHD (Fig. 2A–F). From the t-SNE transformation of hidden layer outputs, we found data points that classified each patient with various prediction scores were randomly distributed after the first encoder layer (Fig. 2A and D). Notably, t-SNE showed that the incorporation of detailed HLA information, even with natural language processing, was not sufficient to differentiate the risk of grade II–IV or III–IV aGVHD (Fig. 2A and D). Moreover, before the training process, concatenating all variables did not permit discrimination of the risk of aGVHD (Fig. 2B and E). However, after the following training process, it was possible to resolve grades II–IV and III–IV aGVHD (Fig. 2C and F). These results suggest that this machine-learning process with an autoencoder and an FC neural network in the CNN model successfully extracted discriminating features.

**Fig. 3:**
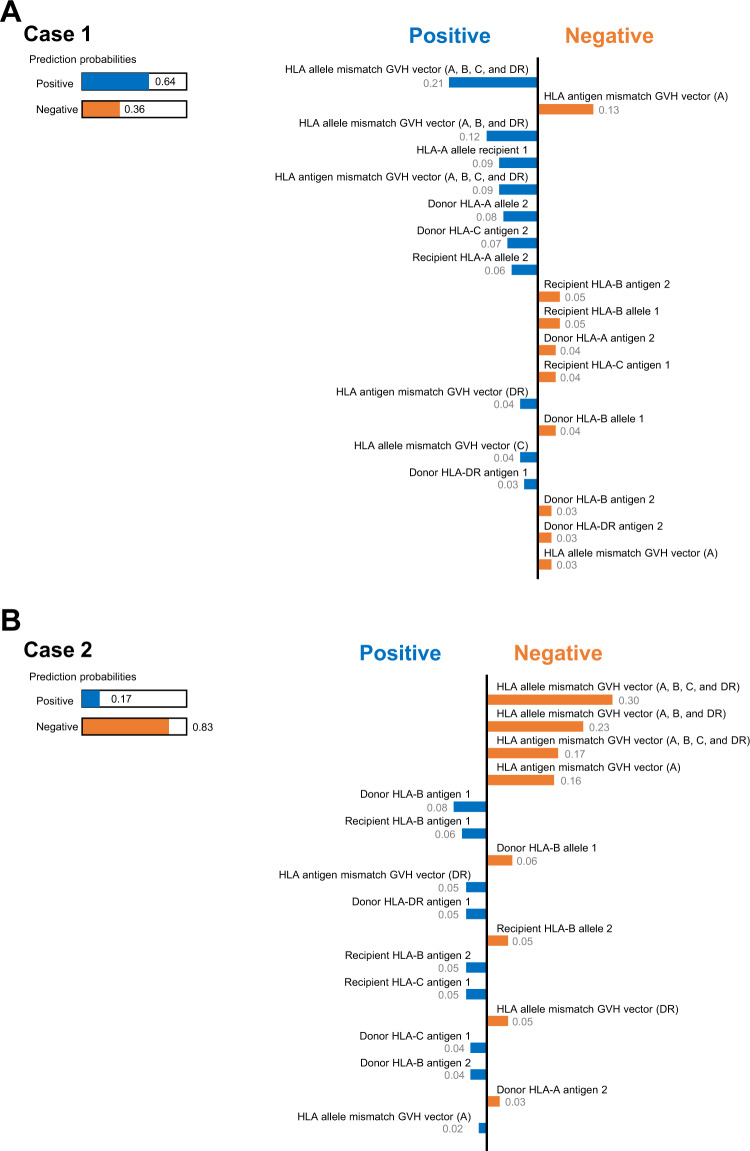
Application of local interpretable model-agnostic explanations (LIME) for two representative cases. **A** For case 1. This example is the case of a relatively higher risk for aGVHD (positive probability of 0.64), including an HLA class I antigen mismatch in the GVH direction. **B** For case 2. This example is the case of relatively lower risk for aGVHD (positive probability of 0.17), including no allelic mismatches in HLA-A, -B, and -DR.

We also utilized local interpretable model-agnostic explanations (LIME) to explain predictions from the CNN algorithm. Representative output examples of LIME analysis, which extracted weights of HLA-related variables, are presented in Fig. 3A and B. In the model output, a positive indicates a probability of developing aGVHD, while a negative indicates that aGVHD is unlikely. Bars indicate the weight of each variable on predictive scores for the risk of aGVHD. Subtraction of these weights from prediction probabilities (1 in both cases as indicated in the left part of the figure) alters the probability of a sample being classified as aGVHD-positive or -negative. The LIME method facilitates the interpretation of factor weighting in the CNN-based predictions of grade II–IV or III–IV aGVHD in individual cases.

### Clinical evaluation of the generalizability of the CNN-based model

**Fig. 4:**
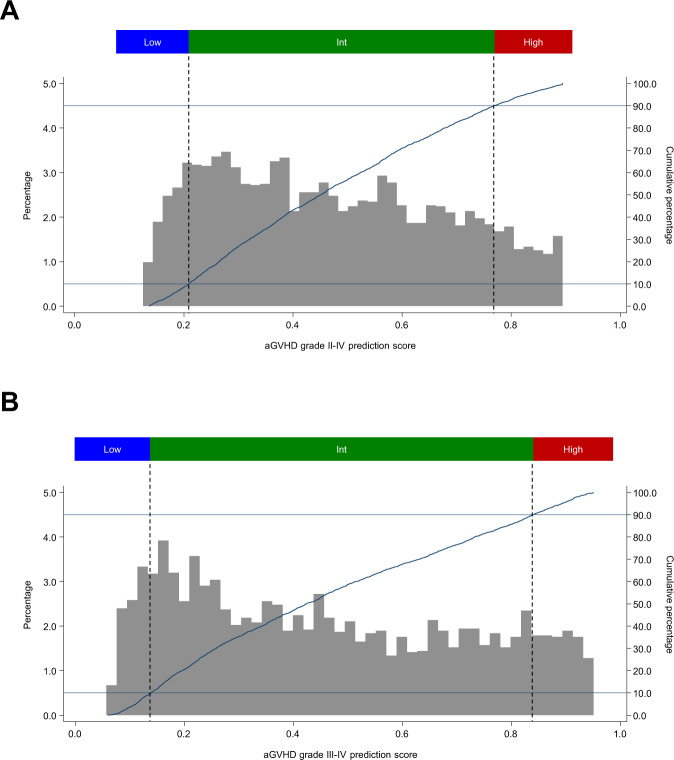
Distribution of acute graft-versus-host disease (aGVHD) predictive scores in the test cohort. Distribution of aGVHD prediction scores calculated by the convolutional neural network (CNN) model are displayed among patients in the test cohort (*N* = 3753). **A** For grade II–IV aGVHD. Low (scores, 0.136–0.209), *n* = 375 (10.0%); Int (scores, 0.209–0.770), *n* = 3003 (80.0%); and High (scores, 0.770–0.894), *n* = 375 (10.0%). **B** For grade III–IV aGVHD. Low (scores, 0.080–0.138), *n* = 375 (10.0%); Int (scores, 0.138–0.840), *n* = 3003 (80.0%); and High (scores, 0840–0.951), *n* = 375 (10.0%). Higher scores indicate a higher risk of developing aGVHD. The data used to plot the graphs is in Supplemental Data 7.

Then, we assessed the generalizability of the developed model using the test cohort. The distribution of prediction scores of grade II–IV and III–IV aGVHD in the test cohort (*n* = 3753) is shown in Fig. 4A and B. For grade II–IV aGVHD, scores ranged from 0.136 to 0.894 (median, 0.450; Fig. 4A). This cohort was divided into three groups according to percentile scores: a low-risk group (Low; 0–10 percentile; range 0.136–0.209; median 0.177; *n* = 375), an intermediate-risk group (Int;10–90 percentile; range 0.209–0.770; median 0.450; *n* = 3003), and a high-risk group (High; 90–100 percentile; range 0.770–0.894; median 0.826; *n* = 375) (Fig. 4A). The distribution of aGVHD III–IV scores is also displayed in Fig. 4B.

**Fig. 5:**
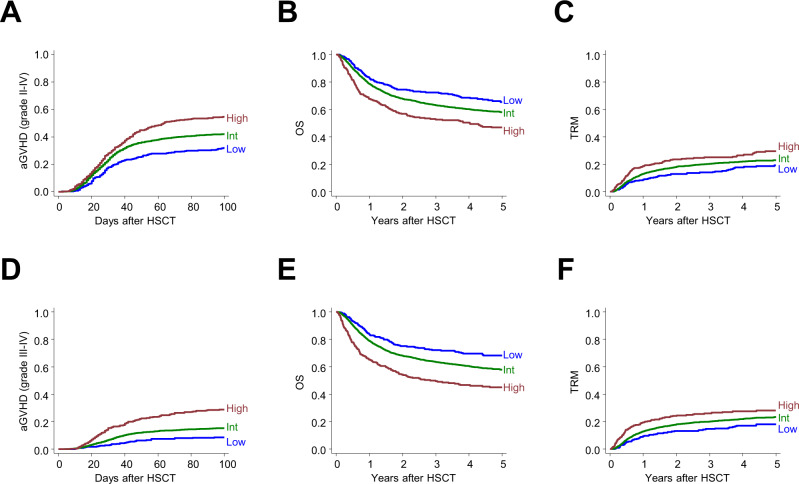
Validation of predictive scores based on the convolutional neural network (CNN) model. **A** Cumulative incidence of grade II–IV acute graft-versus-host disease (aGVHD) in the test cohort is shown according to each risk group for grade II–IV aGVHD (Low, *n* = 375; Int, *n* = 3003; High, *n* = 375). **B** Overall survival (OS) and **C** therapy-related mortality (TRM) were calculated for the same subgroups. **D** Cumulative incidence of grade III–IV aGVHD in the test cohort is shown according to each risk group for grade III–IV aGVHD (Low, *n* = 375; Int, *n* = 3003; High, *n* = 375). **E** OS and **F** TRM were calculated for the same subgroups.

The incidence of aGVHD (for grade II–IV and III–IV) was calculated and compared among the three subgroups for aGVHD risk using conventional statistical techniques (competitive hazard risk models), along with OS and TRM (Fig. 5A–F and Table 1). For grade II–IV, the cumulative incidence of aGVHD was stratified according to each risk group. There was a significantly higher incidence of aGVHD among patients sorted into the High-risk group (54.8% at Day 100) compared with patients in the Low-risk group (31.8% at day 100; hazard ratio [HR], 2.04 vs. Low-risk group; *p* = 0.001) (Fig. 5A and Table 1). OS decreased as the risk of aGVHD increased, probably due to higher incidence of TRM (HR 1.96 and 1.36 in the High-risk vs. Low-risk group, and Intermediate-risk vs. Low-risk group, respectively) (Fig. 5B and C, and Table 1). There was a significant relationship between raw values of prediction scores (continuous variables) and the higher incidence of aGVHD, with higher TRM and inferior OS, calculated using conventional Gray-Fine or Cox proportional-hazard models (Supplemental Data 3). The incidence of grade III–IV aGVHD was also stratified among subgroups (28.8% and 8.4% at Day100 for high and low risk, respectively; HR, 4.02, High-risk vs. Low-risk groups; *p* < 0.001) (Fig. 5D, and Table 1). Grade III–IV GVHD prediction scores were correlated with OS (HR, 1.10 per 0.1) (Fig. 5E, and Supplemental Data 3), most likely as a result of the higher incidence of TRM among higher-risk patients (Fig. 5F). There were also significant relationships between prediction values (raw scores) and higher incidence of aGVHD, higher TRM, and inferior OS (Supplemental Data 3).

**Table 1:** Clinical evaluation for generalizability of the trained model.

Risk group	aGVHD			Overall mortality			TRM		
	HR	95% CI	*P*	HR	95% CI	*P*	HR	95% CI	*P*
Grade II–IV									
Low		Reference			Reference			Reference	
Int	1.44	1.19–1.73	<0.001*	1.36	1.12–1.65	0.002*	1.39	1.07–1.80	0.015*
High	2.04	1.64–2.55	<0.001*	1.96	1.55–2.49	<0.001*	1.81	1.31–2.50	<0.001*
Grade III–IV									
Low		Reference			Reference			Reference	
Int	1.93	1.34–2.78	<0.001*	1.41	1.16–1.72	0.001*	1.38	1.06–1.79	0.016*
High	4.02	2.70–5.97	<0.001*	2.25	1.78–2.86	<0.001*	1.81	1.31–2.51	<0.001*

*aGVHD* acute graft-versus-host disease, *CI* confidence interval, *HR* hazard ratio, *TRM*, transplant-related mortality.

*Indicates *p* < 0.05.

### Performance of the CNN-based model for each subgroup

We then evaluated the performance of the CNN-based model to predict grade II–IV or grade III–IV aGVHD for various patient subgroups (Supplemental Figs. 2 and 3, and Supplemental Data 4 and 5). As a result, we found that the CNN-based model was able to stratify the risk of aGVHD in various patient populations, suggesting that scores calculated with the CNN-based model are applicable in a wide range of clinical settings, regardless of patient background.

### Comprehensive evaluation of factors other than HLA disparity for aGVHD

**Fig. 6:**
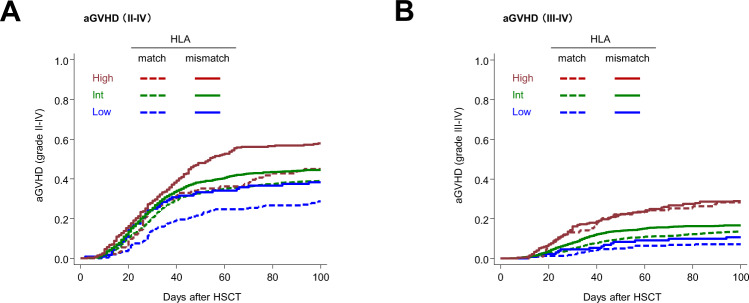
Incidence of acute graft-versus-host disease (aGVHD) according to aGVHD predictive score and HLA disparity. **A** Cumulative incidence of grade II–IV aGVHD in the test cohort is shown according to each risk group for grade II–IV aGVHD and HLA disparity in the test cohort. HLA matched group (*n* = 1763) consisted of Low, *n* = 254; Int, *n* = 1418; and High, *n* = 91. HLA mismatched group (*n* = 1990) consisted of Low, *n* = 121; Int, *n* = 1585; and High, *n* = 284. **B** Cumulative incidence of grade III–IV aGVHD in the test cohort is shown according to each risk group for grade III–IV aGVHD and HLA disparity in the test cohort. HLA matched group (*n* = 1763) consisted of Low, *n* = 240; Int, *n* = 1423; and High, *n* = 100. HLA mismatched group (*n* = 1990) consisted of Low, *n* = 135; Int, *n* = 1580; and High, *n* = 275.

Then, we evaluated the significance of various factors other than HLA disparity for the risk of aGVHD, using the CNN-based model. As expected, prediction scores both of grade II–IV and grade III–IV aGVHD in the HLA-mismatched group were higher than those in the HLA-matched group (Supplemental Fig. 4), reflecting the effects of HLA disparity on the risk of aGVHD. Interestingly, prediction scores based on the current model clearly stratified the risk of grade II–IV and grade III–IV aGVHD both in the HLA-matched and mismatched groups, respectively (Fig. 6A and B). Moreover, patients with high prediction scores in the HLA-matched group had a higher risk of grade II–IV or grade III–IV aGVHD than those with low prediction scores in the HLA-mismatched group. This trend was more pronounced in grade III–IV aGVHD than in grade II–IV acute GVHD. These results suggest that the risk of aGVHD cannot be predicted solely by HLA match/mismatch, but also by a combination of non-HLA parameters. The CNN-based model enabled us to comprehensively evaluate the contributions of various factors to the risk of aGVHD, especially for severe aGVHD.

## Discussion

This machine learning-guided retrospective cohort study investigating risk prediction of aGVHD revealed four major results/findings: (1) A CNN-based model, which can extract discriminating features from comprehensive patient characteristics, was developed to predict risk of aGVHD after HSCT. (2) The learning process employed by the CNN-based model successfully visualized the weight of each clinical factor. (3) Raw HLA data was utilized by the CNN-based model. (4) Influences of factors other than HLA disparity on the risk of aGVHD were clarified.

Whereas CNN made its early success in the area of image analysis, it has also been applied in various areas due to its excellent feature extraction capability^31, 32^. By applying the CNN algorithm, we developed a prediction model for grade II–IV and grade III–IV aGVHD. The generalizability of the model was determined using an internal test cohort. The model succeeded in discriminating between patient groups with a high and low risk of aGVHD. This risk stratification is important because aGVHD is one of the most serious complications, often leading to TRM^33^. Indeed, in this study, patients in the high-risk group had higher TRM and poorer OS than those in the low-risk group (Fig. 5B, C, E, and F). Our reliable prediction model optimizes transplantation procedures by choosing risk-adapted immunosuppression, thereby improving transplantation outcomes.

While the machine learning-based approach has the advantage of unbiased feature selection and prediction, one of the major challenges of machine learning is the difficulty of understanding how it functions. Transparency is essential in order to widely implement a machine-learning model in clinical practice; however, variables used by the model to make its judgment, vary among patients, depending on other clinical factors and interactions between variables. Therefore, these variables must be weighed on a case-by-case basis. For example, HLA disparity, which is a major contributing factor for aGVHD, has different effects among underlying diseases^7^. In this context, our CNN-based model succeeded in visualizing the learning process with t-SNE and in assessing the weights of variables in individual cases using LIME. Our results suggest that this CNN-based model employing techniques that render it transparent and comprehensible will help clinicians to select optimal donor sources and transplantation procedures with confidence.

While we and other groups have previously developed machine learning-based models to predict outcomes after HSCT^7–9^, but the arbitrariness of variable settings has not been solved, especially regarding HLA information. In this study, for the first time, we incorporated raw information about specific antigens and/or alleles of both donors and recipients into a machine learning-based prediction model. Previous studies with conventional linear proportional hazard models or machine-learning models treated HLA information as binary data (matched or mismatched). However, the degree of HLA disparity may not be equivalent depending on combinations of specific HLA antigens and/or alleles between donor and recipient. For example, the difference between HLA-A02:01 and HLA-A02:02 may not always be the same as that between HLA-A02:01 and HLA-A11:01. We successfully imported raw HLA information in the CNN-based model by utilizing word2vec, a natural-language processing method. While this study did not identify novel HLA combinations that consistently alter the risk of developing aGVHD irrespective of patient background, visualization efforts using LIME enabled us to assess the contributions of HLA antigens or alleles of donors and recipients to the risk of aGVHD in individual cases. Machine-learning models that combine biological HLA information, including epitopes and molecular structures, in a larger cohort may provide further detailed information about the contributions of specific combinations of HLA antigens or alleles to aGVHD risk^6^.

In this study, we clarified the impact of factors other than HLA disparity on the risk of aGVHD using a CNN-based model. HLA mismatching is the most important risk factor for acute GVHD, but effects of other clinical factors on the risk of aGVHD have differed from report to report due to differences in patient characteristics^4^, and the contributions of these factors other than HLA disparity have not been fully evaluated. This study revealed contributions of clinical factors other than HLA mismatches to the risk of aGVHD. In this study, we found that there was a group even among patients transplanted from HLA-matched donors who were at a higher risk of developing aGVHD than those transplanted from HLA-mismatched donors and another group among those transplanted from HLA-mismatched donors who had an extremely low risk of severe aGVHD. While this study showed that individual patients had a different weight for each factor in the risk of developing acute GVHD, a comparison of patients who had HLA-matched and the highest prediction scores for aGVHD II–IV (matched highest group) and those who had HLA-mismatched and the lowest prediction scores (mismatched lowest group) revealed that matched highest group patients tended to be older, more male, worse performance status, have more frequent complications of major organ, use more RIC, and use less MTX and MMF for GVHD prophylaxis than the entire cohort, and that mismatched lowest group patients tended to be younger, use less peripheral blood stem cells as graft sources, and use less ATG than the entire cohort (Supplemental Data 6). Thus, prophylactic measures to reduce the risk of aGVHD should be optimized according to comprehensive prediction models that incorporate various clinical factors, rather than depending solely on HLA matching.

The present study revealed the utility of CNN as a prognostic tool for aGVHD. However, there are some limitations to this study that must be addressed. While the CNN model was designed to avoid researcher bias in variable settings, some of the variables were categorized into subgroups based on clinically established criteria. For example, we stratified pre-transplant disease conditions using disease risk. Another limitation is that our outcome measure, the incidence of aGVHD, was also treated as a binary variable in the CNN-based model that we used in this study. Information on the onset time for cases of aGVHD was not included in the process of model development. In this study, the onset of aGVHD is limited to a small window (usually 30–100 days after HSCT); therefore, the effect of ignoring information regarding the time of onset is probably suboptimal. Because biological HLA information was not included in this study, the risk of aGVHD can potentially be affected by combinations of HLA alleles that are different in notation, but are biologically homologous. In this study, we included as many variables accessible and consistently evaluable in the existing registry for the establishment of prediction models but might ignore the potential effects of unavailable parameters on the risk of acute GVHD. And the inclusion of parameters early after transplantation in addition to pre-transplant factors can improve the stratification power of the model, as previously reported^34^. While technical improvements are required to collect information on a larger number of parameters, incorporating more parameters, including variables with uncertain significance at present, into the machine learning model is beneficial to maximize the potential of machine learning. In this study, missing values regarding several variables were handled by the model, missingness can potentially affect the prediction. Overfitting is the conventionally discussed limitation in machine learning^35^, and our algorithm is not completely free of this limitation, even though we took measures to avoid it. In addition, ethnicity affects the incidence and severity of GVHD^36^. While the main architecture of our model can be applied to various different cohorts, tuning the model is required to apply this model in different cohorts. Therefore, further validation of the CNN-based model using different cohorts, including other ethnic groups, by using our approach as a proof-of-concept is needed. We note that alternative machine learning algorithms, such as random forest regression and recurrent neural networks, have seen increased application to problems with clinical practices in recent years, and maybe equally suited to CNN-based models, and the optimal machine learning approach should be further studied.

In conclusion, we developed a CNN-based prediction model for aGVHD after allogeneic HSCT using a nationwide transplant database in Japan, which incorporates comprehensive HLA information, excluding arbitrariness, as well as ensuring transparency of the calculation process. This prediction model revealed that the risk of aGVHD is determined not only by HLA disparity but also by detailed HLA information, as well as various clinical factors other than HLA. This study suggests that our CNN-based prediction model can be used to establish various prognostic predictive models in the field of HSCT, which is applicable in clinical practice.

## Supplementary information

Description of Additional Supplementary Files

Supplementary Information

Supplementary Data 1

Supplementary Data 2

Supplementary Data 3

Supplementary Data 4

Supplementary Data 5

Supplementary Data 6

Supplementary Data 7

Supplementary Data 8

Supplementary Data 9

Reporting Summary

## Data Availability

The data that supports the findings of this study are not openly available due to reasons of sensitivity (patient privacy) and are available from the corresponding author upon reasonable request such as the application of novel drugs. The data used to plot the graphs in Fig. 4 is in Supplemental Data 7.

## Code availability

The code that supports the findings of this study is in Supplemental Data 8 and 9.
