# Author Correction: A convolutional neural network-based model that predicts acute graft-versus-host disease after allogeneic hematopoietic stem cell transplantation

**DOI:** 10.1038/s43856-023-00323-8

**Published:** 2023-06-22

**Authors:** Tomoyasu Jo, Yasuyuki Arai, Junya Kanda, Tadakazu Kondo, Kazuhiro Ikegame, Naoyuki Uchida, Noriko Doki, Takahiro Fukuda, Yukiyasu Ozawa, Masatsugu Tanaka, Takahide Ara, Takuro Kuriyama, Yuta Katayama, Toshiro Kawakita, Yoshinobu Kanda, Makoto Onizuka, Tatsuo Ichinohe, Yoshiko Atsuta, Seitaro Terakura

**Affiliations:** 1 Department of Hematology and Oncology, Graduate School of Medicine, Kyoto University, Kyoto, Japan; 2 Center for Research and Application of Cellular Therapy, Kyoto University Hospital, Kyoto, Japan; 3 Department of Hematology, Hyogo Medical University Hospital, Hyogo, Japan; 4 Department of Hematology, Federation of National Public Service Personnel Mutual Aid Associations Toranomon Hospital, Tokyo, Japan; 5 Hematology Division, Tokyo Metropolitan Cancer and Infectious Diseases Center, Komagome Hospital, Tokyo, Japan; 6 Department of Hematopoietic Stem Cell Transplantation, National Cancer Center Hospital, Tokyo, Japan; 7 Department of Hematology, Japanese Red Cross Aichi Medical Center Nagoya Daiichi Hospital, Nagoya, Japan; 8 Department of Hematology, Kanagawa Cancer Center, Yokohama, Japan; 9 Department of Hematology, Hokkaido University Hospital, Sapporo, Japan; 10 Department of Hematology, Hamanomachi Hospital, Fukuoka, Japan; 11 Department of Hematology, Hiroshima Red Cross Hospital & Atomic-bomb Survivors Hospital, Hiroshima, Japan; 12 Department of Hematology, National Hospital Organization Kumamoto Medical Center, Kumamoto, Japan; 13 Division of Hematology, Jichi Medical University Saitama Medical Center, Saitama, Japan; 14 Department of Hematology/Oncology, Tokai University School of Medicine, Isehara, Japan; 15 Department of Hematology and Oncology, Research Institute for Radiation Biology and Medicine, Hiroshima University, Hiroshima, Japan; 16 Japanese Data Center for Hematopoietic Cell Transplantation, Nagoya, Japan; 17 Department of Registry Science for Transplant and Cellular Therapy, Aichi Medical University School of Medicine, Nagakute, Japan; 18 Department of Hematology and Oncology, Nagoya University Graduate School of Medicine, Nagoya, Japan

**Keywords:** Haematopoietic stem cells, Computational biology and bioinformatics, Haematological diseases, Graft-versus-host disease

Correction to: *Communications Medicine* 10.1038/s43856-023-00299-5, published online 16 May 2023.

In this article the funding from Novartis Pharma was omitted. The original article has been corrected.

