# Supplementary material for: A convolutional neural network-based model that predicts acute graft-versus-host disease after allogeneic hematopoietic stem cell transplantation
Source: Commun Med (Lond). 2023 May 16;3:67. doi: 10.1038/s43856-023-00299-5 (PMC10188562; doi:10.1038/s43856-023-00299-5)
Supplement: Supplementary file 1 — Description of Additional Supplementary Files [file 43856_2023_299_MOESM1_ESM.pdf]

Description of Additional Supplementary File

File Name: Supplementary Data 1

Description: Variable lists considered in the predictive models

File Name: Supplementary Data 2

Description: Patient characteristics in the training, validation and test cohorts

File Name: Supplementary Data 3

Description: Validation analysis of predictive scores

File Name: Supplementary Data 4

Description: Number of patients in each risk group among subgroups in the test cohort

File Name: Supplementary Data 5

Description: Validation analysis of predictive score as per subgroup

File Name: Supplementary Data 6

Description: Patient characteristics among patients who have HLA matched and the highest prediction scores and those who have HLA mismatched and the lowest prediction scores.

File Name: Supplementary Data 7

Description: The data used to plot the graphs in Figure 4

File Name: Supplementary Data 8

Description: The code that support the building of the CNN model

File Name: Supplementary Data 9

Description: The code that support the validation of the CNN model
