# Supplementary material for: A convolutional neural network-based model that predicts acute graft-versus-host disease after allogeneic hematopoietic stem cell transplantation
Source: Commun Med (Lond). 2023 May 16;3:67. doi: 10.1038/s43856-023-00299-5 (PMC10188562; doi:10.1038/s43856-023-00299-5)
Supplement: Supplementary file 2 — Supplementary Information [file 43856_2023_299_MOESM2_ESM.pdf]

Supplemental Figure 1

A

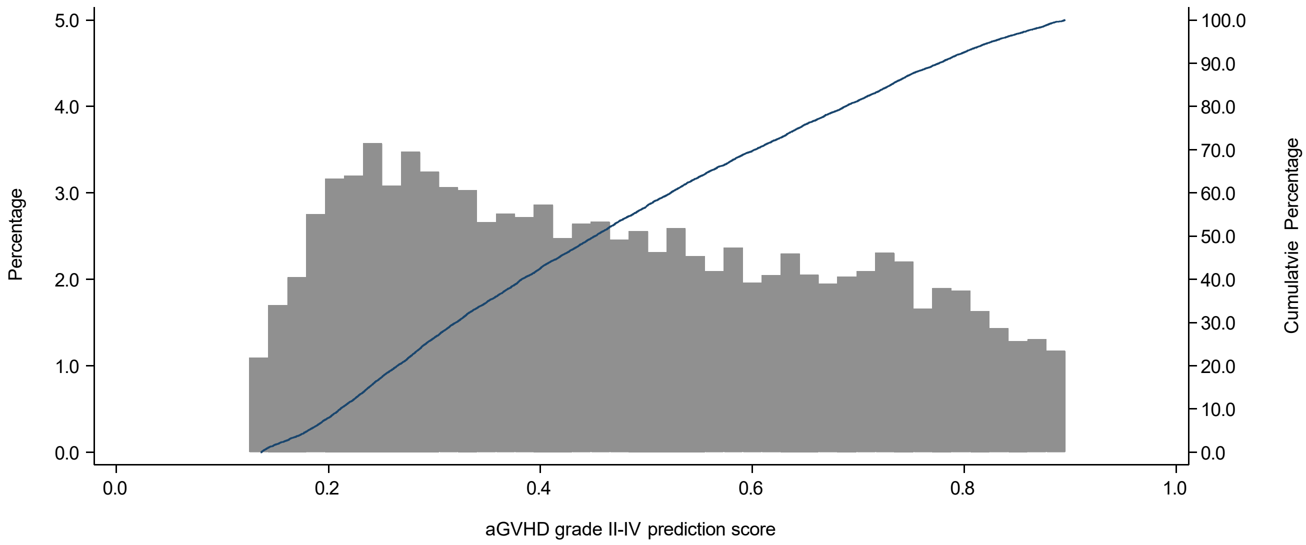

B

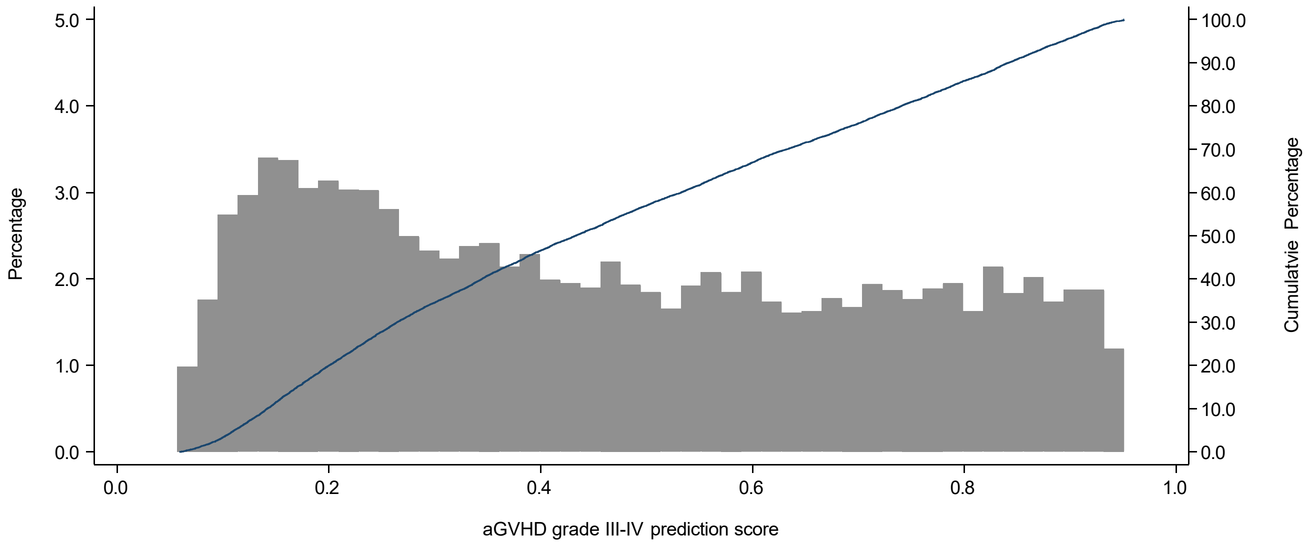

Supplemental Figure 1. Distribution of acute graft-versus-host disease (aGVHD) predictive scores in the training cohort. Distribution of aGVHD predictive scores calculated by the convolutional neural network (CNN) model are displayed among patients in the training cohort. **(A)** For grade II–IV aGVHD. **(B)** For grade III–IV aGVHD. Higher scores indicate a higher risk of developing aGVHD.

Supplemental Figure 2

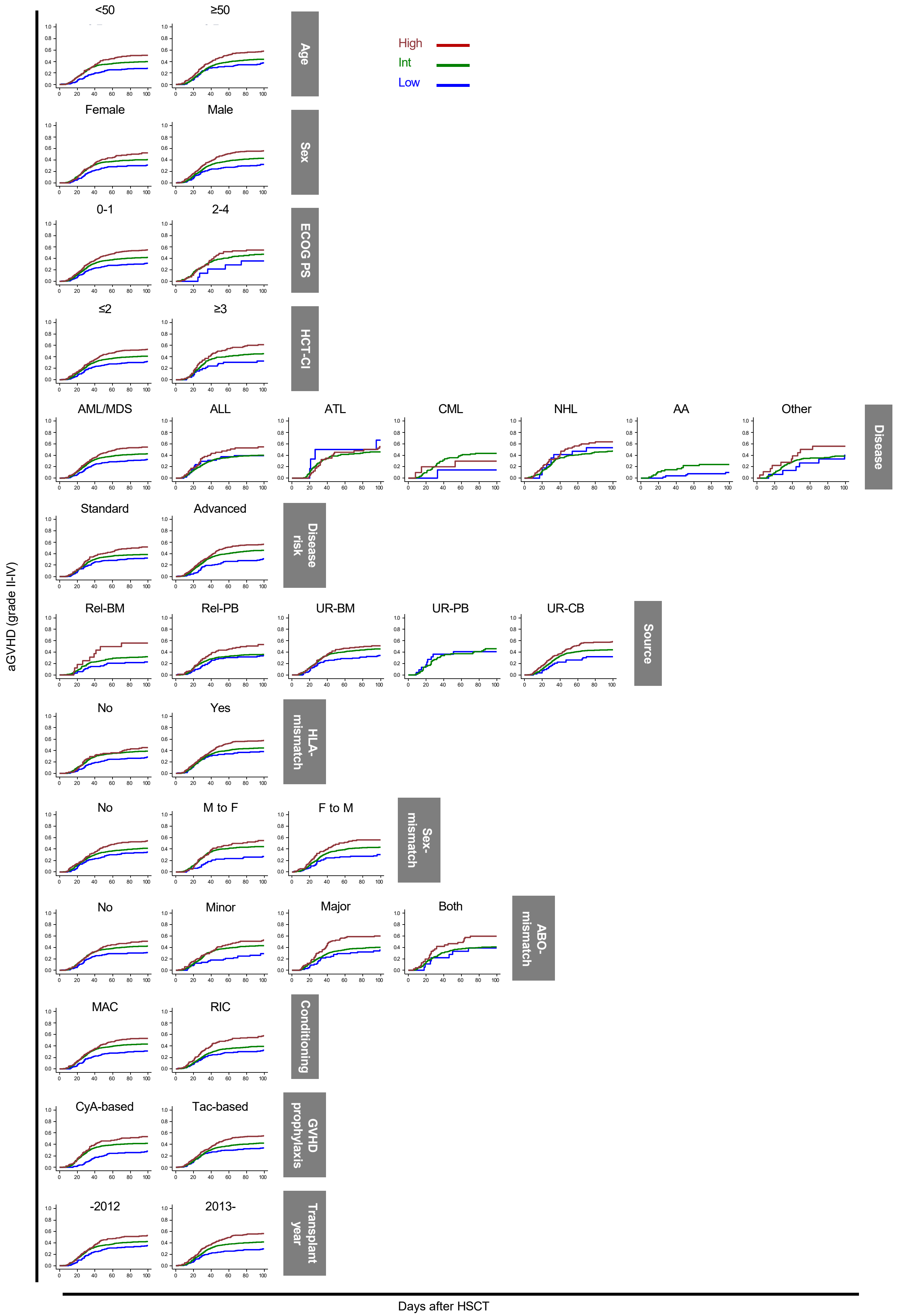

Supplemental Figure 2. Performance of the convolutional neural network (CNN)-based model of grade II–IV aGVHD for each subgroup.

Supplemental Figure 3

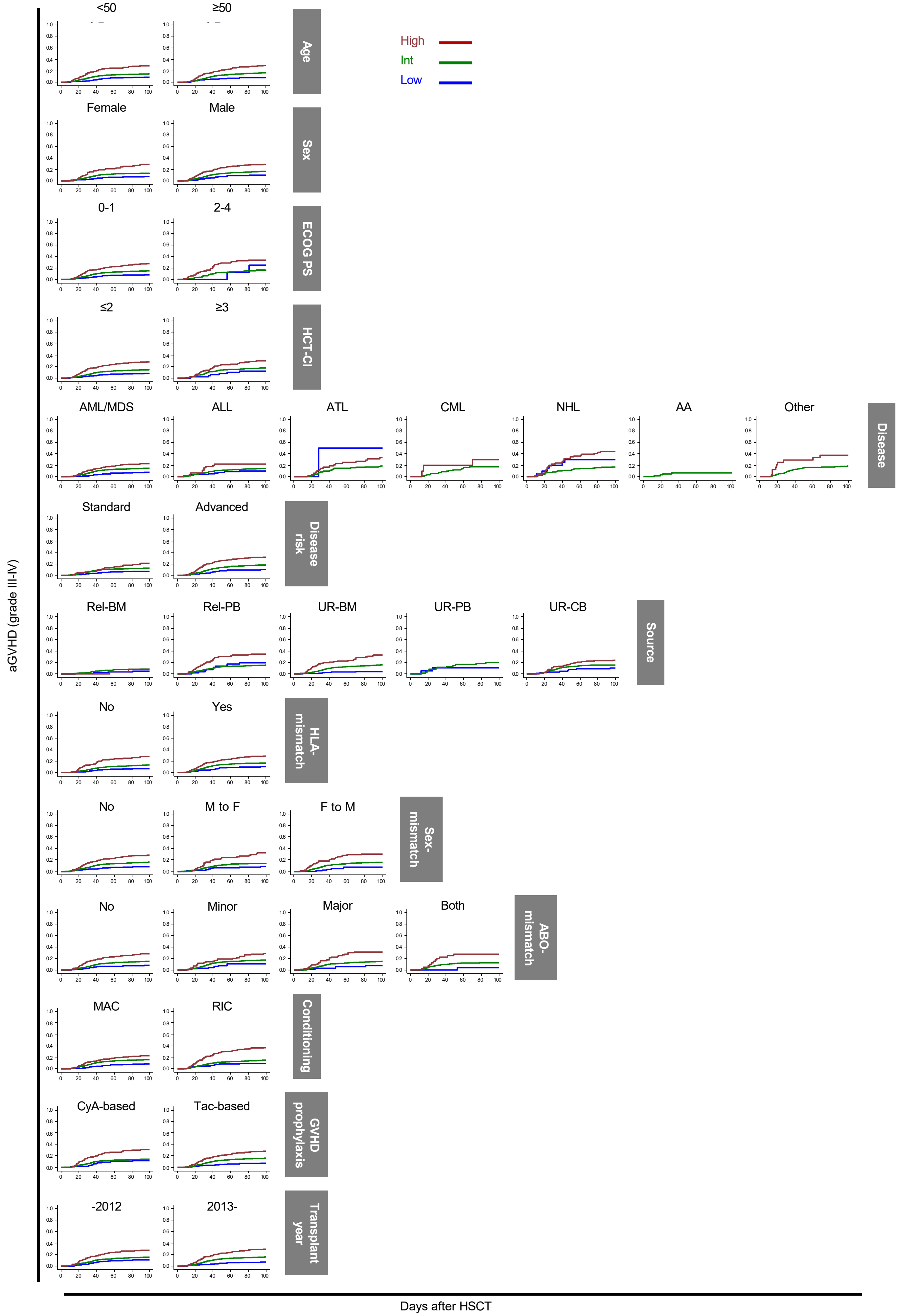

Supplemental Figure 3. Performance of the convolutional neural network (CNN)-based model of grade III–IV aGVHD for each subgroup.

Supplemental Figure 4

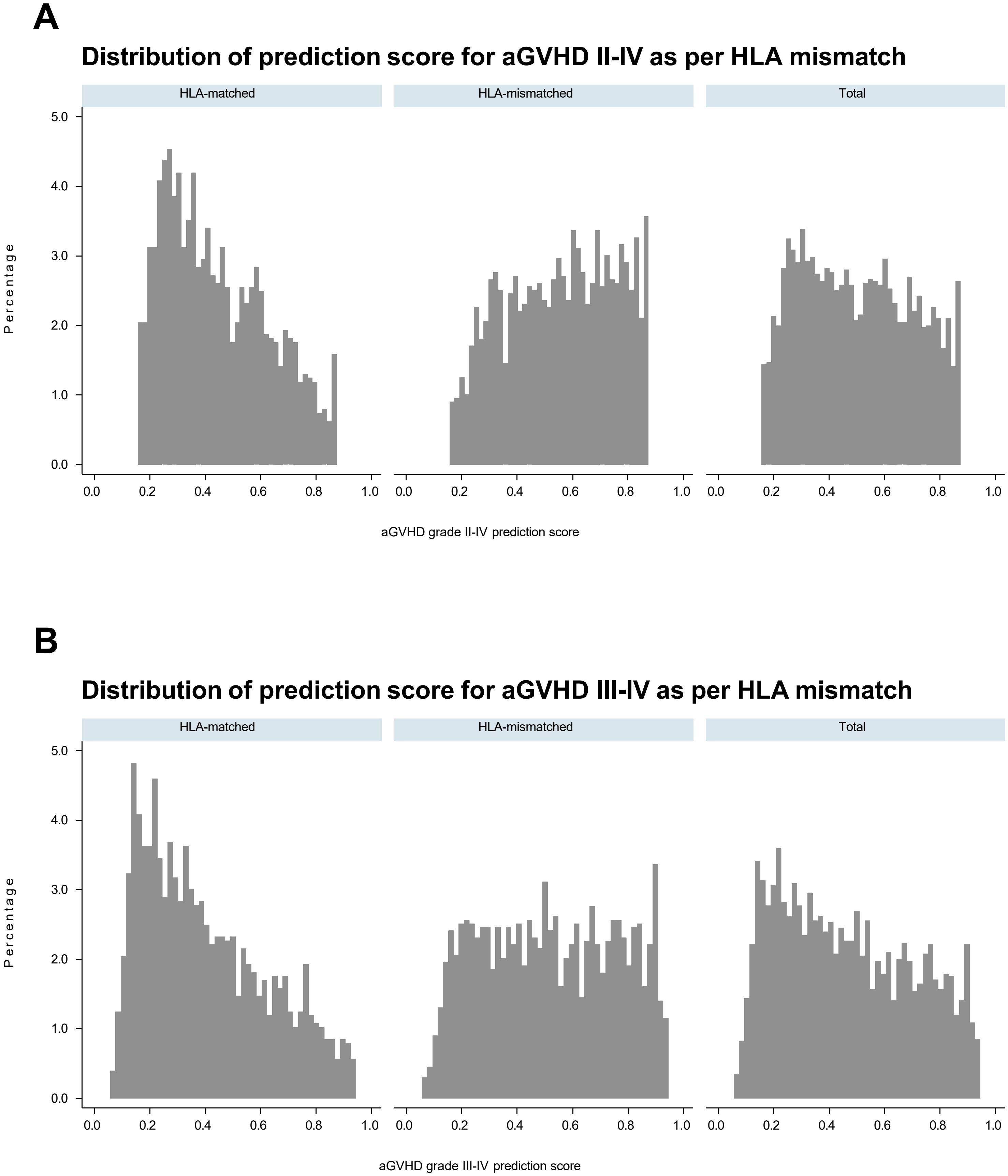

Supplemental Figure 4. Distribution of acute graft-versus-host disease (aGVHD) predictive scores in the test cohort according to HLA disparity. (A) For grade II–IV aGVHD. **(B)** For grade III–IV aGVHD.
