# Supplementary material for: A convolutional neural network-based model that predicts acute graft-versus-host disease after allogeneic hematopoietic stem cell transplantation
Source: Commun Med (Lond). 2023 May 16;3:67. doi: 10.1038/s43856-023-00299-5 (PMC10188562; doi:10.1038/s43856-023-00299-5)
Supplement: Supplementary file 12 — Reporting Summary [file 43856_2023_299_MOESM12_ESM.pdf]

nature portfolio

Corresponding author(s): Yasuyuki Arai

Last updated by author(s): Apr 26, 2023

Reporting Summary

Nature Portfolio wishes to improve the reproducibility of the work that we publish. This form provides structure for consistency and transparency in reporting. For further information on Nature Portfolio policies, see our Editorial Policies and the Editorial Policy Checklist.

Statistics

For all statistical analyses, confirm that the following items are present in the figure legend, table legend, main text, or Methods section.

n/a	Confirmed	
☐	☒	The exact sample size (n) for each experimental group/condition, given as a discrete number and unit of measurement
☐	☒	A statement on whether measurements were taken from distinct samples or whether the same sample was measured repeatedly
☐	☒	The statistical test(s) used AND whether they are one- or two-sided<br>*Only common tests should be described solely by name; describe more complex techniques in the Methods section.*
☐	☒	A description of all covariates tested
☐	☒	A description of any assumptions or corrections, such as tests of normality and adjustment for multiple comparisons
☐	☒	A full description of the statistical parameters including central tendency (e.g. means) or other basic estimates (e.g. regression coefficient) AND variation (e.g. standard deviation) or associated estimates of uncertainty (e.g. confidence intervals)
☐	☒	For null hypothesis testing, the test statistic (e.g. F, t, r) with confidence intervals, effect sizes, degrees of freedom and P value noted<br>*Give P values as exact values whenever suitable.*
☒	☐	For Bayesian analysis, information on the choice of priors and Markov chain Monte Carlo settings
☐	☒	For hierarchical and complex designs, identification of the appropriate level for tests and full reporting of outcomes
☐	☒	Estimates of effect sizes (e.g. Cohen's d, Pearson's r), indicating how they were calculated

Our web collection on statistics for biologists contains articles on many of the points above.

Software and code

Policy information about availability of computer code

Data collection	Data on adult patients (age ≥ 16 years) with malignant and nonmalignant hematological diseases who underwent allogeneic HSCT between 1 January 2008 and 31 December 2018 were obtained from the Japanese Transplant Registry Unified Management Program sponsored by the Japanese Society for Transplantation and Cellular Therapy (JSTCT) and the Japanese Data Center for Hematopoietic Cell Transplantation.
Data analysis	The CNN architecture was implemented in Python using the Keras library, which is a high-level library for TensorFlow version 2.2 ML framework. Stata (version 17; Stata Corp., College Station, TX) was used to analyze data.

For manuscripts utilizing custom algorithms or software that are central to the research but not yet described in published literature, software must be made available to editors and reviewers. We strongly encourage code deposition in a community repository (e.g. GitHub). See the Nature Portfolio guidelines for submitting code & software for further information.

Data

Policy information about availability of data

All manuscripts must include a data availability statement. This statement should provide the following information, where applicable:

- Accession codes, unique identifiers, or web links for publicly available datasets
- A description of any restrictions on data availability
- For clinical datasets or third party data, please ensure that the statement adheres to our policy

The data that supports the findings of this study is not openly available due to reasons of sensitivity (privacy of patients) and are available from the corresponding author upon reasonable request.

Research involving human participants, their data, or biological material

Policy information about studies with human participants or human data. See also policy information about sex, gender (identity/presentation), and sexual orientation and race, ethnicity and racism.

Reporting on sex and gender	Sex of patients and donors along with sex mismatched between patients and donors are significant prognostic factors in transplantation. Therefore, reporting on sex is included in this study.
Reporting on race, ethnicity, or other socially relevant groupings	Not used
Population characteristics	Data on adult patients (age ≥ 16 years) with malignant and nonmalignant hematological diseases who underwent allogeneic HSCT between 1 January 2008 and 31 December 2018 in Japan is included.
Recruitment	With government support, Japanese Society for Transplantation and Cellular Therapy (JSTCT) and the Japanese Data Center for Hematopoietic Cell Transplantation (JDCHCT) is responsible for collecting and analyzing HSCT recipient and donor information based on the "Act for Appropriate Provision of Hematopoietic Stem Cells to be Used in Transplantations."
Ethics oversight	Kyoto University, Kyoto, Japan

Note that full information on the approval of the study protocol must also be provided in the manuscript.

Field-specific reporting

Please select the one below that is the best fit for your research. If you are not sure, read the appropriate sections before making your selection.

☒ Life sciences ☐ Behavioural & social sciences ☐ Ecological, evolutionary & environmental sciences

For a reference copy of the document with all sections, see nature.com/documents/nr-reporting-summary-flat.pdf

Life sciences study design

All studies must disclose on these points even when the disclosure is negative.

Sample size	18,763 patients
Data exclusions	Patients were excluded if information about HLA mismatch, aGVHD grade, or clinical outcomes (dead or alive) were missing.
Replication	All attempts at replication were all successful.
Randomization	Not randomized according to the nature of retrospective cohort study.
Blinding	Not blinded according to the nature of retrospective cohort study.

Reporting for specific materials, systems and methods

We require information from authors about some types of materials, experimental systems and methods used in many studies. Here, indicate whether each material, system or method listed is relevant to your study. If you are not sure if a list item applies to your research, read the appropriate section before selecting a response.

Materials & experimental systems

n/a	Involved in the study	
☒	☐	Antibodies
☒	☐	Eukaryotic cell lines
☒	☐	Palaeontology and archaeology
☒	☐	Animals and other organisms
☐	☒	Clinical data
☒	☐	Dual use research of concern
☒	☐	Plants

Methods

n/a	Involved in the study	
☒	☐	ChIP-seq
☒	☐	Flow cytometry
☒	☐	MRI-based neuroimaging

Clinical data

Policy information about clinical studies

All manuscripts should comply with the ICMJE guidelines for publication of clinical research and a completed CONSORT checklist must be included with all submissions.

Clinical trial registration	Registered to the research program under the Japanese Data Center for Hematopoietic Cell Transplantation.
Study protocol	the Japanese Data Center for Hematopoietic Cell Transplantation
Data collection	Data on adult patients (age ≥ 16 years) with malignant and nonmalignant hematological diseases who underwent allogeneic HSCT between 1 January 2008 and 31 December 2018 were obtained from the Japanese Transplant Registry Unified Management Program sponsored by the Japanese Society for Transplantation and Cellular Therapy (JSTCT) and the Japanese Data Center for Hematopoietic Cell Transplantation.
Outcomes	Primary end-point is the incidence of aGVHD; secondary overall survival
